# A Biomimetic Pose Estimation and Target Perception Strategy for Transmission Line Maintenance UAVs

**DOI:** 10.3390/biomimetics9120745

**Published:** 2024-12-06

**Authors:** Haoze Zhuo, Zhong Yang, Chi Zhang, Nuo Xu, Bayang Xue, Zekun Zhu, Yucheng Xie

**Affiliations:** 1 College of Automation Engineering, Nanjing University of Aeronautics and Astronautics, Nanjing 211106, China; zhuohaoze@nuaa.edu.cn (H.Z.); xunuo@nuaa.edu.cn (N.X.); zachary@nuaa.edu.cn (Z.Z.); chengyx@nuaa.edu.cn (Y.X.); 2 Electric Power Research Institute of Guangxi Power Grid Co., Ltd., Nanning 530000, China

**Keywords:** biomimetic, SLAM, transmission line inspection, intelligent robots, target tracking

## Abstract

High-voltage overhead power lines serve as the carrier of power transmission and are crucial to the stable operation of the power system. Therefore, it is particularly important to detect and remove foreign objects attached to transmission lines, as soon as possible. In this context, the widespread promotion and application of smart robots in the power industry can help address the increasingly complex challenges faced by the industry and ensure the efficient, economical, and safe operation of the power grid system. This article proposes a bionic-based UAV pose estimation and target perception strategy, which aims to address the lack of pattern recognition and automatic tracking capabilities of traditional power line inspection UAVs, as well as the poor robustness of visual odometry. Compared with the existing UAV environmental perception solutions, the bionic target perception algorithm proposed in this article can efficiently extract point and line features from infrared images and realize the target detection and automatic tracking function of small multi-rotor drones in the power line scenario, with low power consumption.

## 1. Introduction

Electricity is one of the main energy sources required for industrial production and human life, and the demand for electric power is enormous in all countries around the world. Power grid facilities are not only widely distributed and span multiple regions, but also operate in highly complex environments, so the safe operation and maintenance of the power grid is extremely important. High-voltage transmission lines are mostly exposed metal conductors and once foreign objects become attached to them, such as trees, kites, balloons, and plastic bags, they are very likely to cause power line short circuits, seriously threatening the safe operation of the transmission system. Therefore, regularly removing foreign objects attached to high-voltage power lines and tree obstacles near transmission conductors are important tasks in power grid maintenance. Especially in hilly or mountainous areas, foreign object removal has become a major work-related difficulty and pain point for power grid maintenance personnel. However, relying solely on traditional manual operation to undertake power grid maintenance work requires a large number of personnel and current power maintenance departments have far fewer personnel working for them than the actual power assurance needs. Therefore, the traditional manual obstacle removal method for high-voltage transmission lines has the disadvantages of posing high safety risks for personnel and low efficiency. Intelligent UAVs involved in power operations, which offer advantages including rapid deployment and flexible portability, are undoubtedly a safe and economical alternative to manual maintenance [1,2,3].

In recent years, there have been many pieces of research on the use of aerial robots to replace manual work in regard to power grid maintenance and some have been successfully applied [4,5,6], as shown in Table 1. The work in [4] involved the design of an aerial robot for power line inspection based on a quadrotor drone, using wireless command transmission and visual image technology. The authors created detailed designs, from the overall control scheme to the subsystems, and conducted actual flight tests, with the experimental results proving the correctness and effectiveness of the control scheme. The study in [6] uses image processing and ultrasonic technology to design a multi-rotor line patrol aerial robot. The authors created detailed designs, from the overall control scheme to the system hardware and software, and organized actual machine tests, with the results verifying the practicality of the design work.

Unfortunately, the existing UAV pose estimation and environmental perception methods still face huge challenges in regard to actual power line maintenance. On the one hand, due to the limited payload and energy reserve of small drones, it is difficult to adapt large-volume and high-power on-board sensors, and the computing power of the drone’s on-board computer is far lower than desktop computers, which leads directly to many high-performance algorithms performing very poorly during actual transmission line maintenance. On the other hand, the performance of the drone’s pose estimator is significantly degraded due to factors such as body shaking, lighting changes, texture deficiency, dynamic target interference, and multipath effects during the flight process and erroneous pose estimation results will affect the environmental perception effect of the drone, causing distortion and deformation of the scene map around the transmission lines. In addition, the integration of drones with current advanced artificial intelligence technologies is still not mature, making it difficult to generate a multi-level semantic understanding of the environment where the drone is located and meaning that such systems are unable to meet the requirements of advanced power operation tasks.

To overcome the urgent scientific problems mentioned above, this article turns the focus to the natural world, in order to seek new design inspiration from animals in nature. The retina of owls usually contains a large number of rod cells, which have the ability to navigate and locate in low-light environments at night. Unlike cone cells, rod cells are specialized optic nerve cells in the retina that are used to sense weak light stimuli. They are not sensitive to color reactions and are highly sensitive to changes in light intensity. Owls and other nocturnal animals have a higher proportion of rod cells in their retina, making their visual perception more sensitive during nighttime activities compared to other animals. Owls can use the shear cells in their visual system to compare the brightness changes in the retina, thereby perceiving the motion information of visual features, and estimate their own pose based on the changes to the visual features in the retina [7,8,9,10,11]. By representing the pixel motion in the image as vector information, the change to visual features can reflect spatial motion information, such as object displacement and attitude change. The change to visual features on the retina can be considered as the projection of the three-dimensional motion of objects onto their two-dimensional imaging plane; therefore, using the change to the visual features in the image to estimate the pose requires the inference of the magnitude and direction of the optical flow from the pixel gradients in the image. Unlike traditional pose estimation methods, visual feature matching and tracking is a biologically inspired pose estimation method, which realizes the tracking of feature points in the image by simulating the mechanism of shear cell processing of retinal brightness changes.

**Figure 1:**
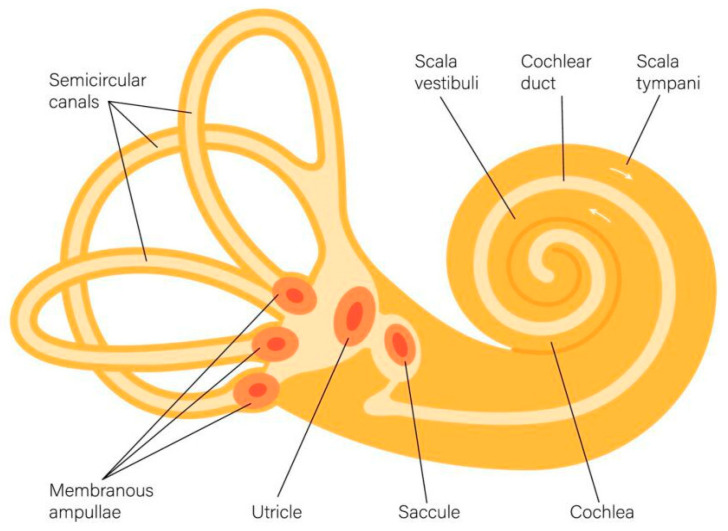
Structural diagram of vestibular organs in vertebrates.

In addition to the visual system, the inner labyrinth is also an important sensory organ in animals. As shown in Figure 1, the inner labyrinth in vertebrates, in addition to the cochlea, which is sensitive to hearing, also includes three organs: semicircular canals, the utricle, and the sacculus, which together constitute the vestibular system [12,13,14]. Semicircular canals can measure the rotational motion of the head, while the utricle and saccule can sense the head’s acceleration motion, including gravity. The vestibular organ is the sensory organ in vertebrates involved in their current motion state and spatial position, and when the animal performs linear acceleration or rotation, the forces generated by the acceleration motion will stimulate the vestibular ganglion cells distributed in the semicircular canals or utricle. These stimuli will cause neural impulses that propagate along the eighth cranial nerve to the central nervous system, thereby causing corresponding sensations of motion. Therefore, the vestibular organ is considered the main organ for measuring biological spatial perception.

In recent years, researchers have designed a new algorithm to track signal propagation in the multi-synaptic pathways in the bird brain and have analyzed the functions of feedforward and feedback pathways, multi-sensory integration, and cross-hemispheric responses [15,16,17,18,19,20]. It was found that the bird brain has a highly recurrent neural structure, with a unit similar to an attention mechanism for information processing. The visual center of an owl has different degrees of visual information perception efficiency, i.e., visual acuity, and the central fovea region has the strongest visual acuity. In order to maximize the information processing efficiency of the visual nervous center, an owl’s brain will adaptively select the most interesting part of the visual area (such as food, predators, etc.) and ignore irrelevant information. When the owl’s own target detection and tracking capabilities are limited, the attention mechanism is the main means to solve the information overload problem, and this ability can allocate the owl’s limited resources to what is currently the most important task.

**Figure 2:**
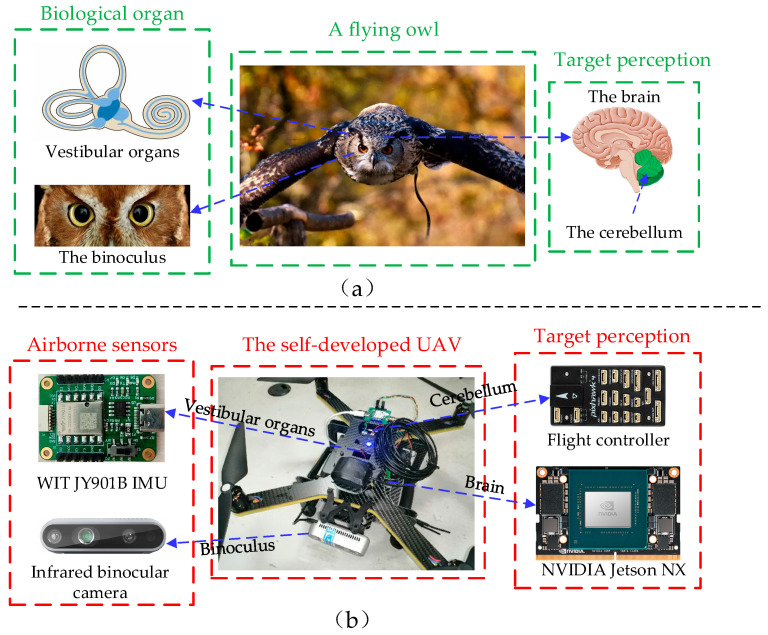
Comparison of environmental perception mechanism in an owl and a UAV: (**a**) owl pose estimation and environmental perception module; (**b**) the pose estimation and environmental perception system in a quad-rotor UAV.

Drawing inspiration from the multi-modal pose estimation and environmental perception mechanisms of wild owls, this article proposes a novel visual–inertial pose estimation method, based on the fusion of point and line features, for power line inspection drones, as shown in Figure 2. Due to the abundance of power line features in power line scenarios, by fusing the corner features and line features in infrared images, and using a point–line fusion-based loop-closure detection module to relocalize the UAV, a new visual–inertial pose estimation system is proposed. Compared with traditional visual odometry methods, the proposed algorithm can efficiently extract point and line features from infrared images and has stronger robustness in low-texture and abrupt lighting environments. In addition, to address the lack of pattern recognition and automatic tracking capabilities of the current power line inspection drones, this article proposes a lightweight neural network-based target detection and tracking strategy. Compared to existing target detectors, the target tracking algorithm proposed in this article can enable the functioning of target detection and automatic tracking by a small multi-rotor UAV in power line scenarios, with low power consumption. The innovative research detailed in this article is as follows:

- Inspired by an owl’s binoculus and vestibular organs, a novel UAV pose estimation method is designed, based on the multi-modal comprehensive pose perception mechanism in owls, using a binocular camera and the inertial measurement unit (IMU) to simulate an owl’s retina and vestibular organs, respectively, enabling the UAV to acquire autonomous navigation capabilities similar to animals;
- Inspired by an owl’s visual attention mechanism, a lightweight bionic neural network, OVNet (owl vision net), is proposed based on the visual nervous system in owls. A visual depth estimation algorithm, based on the binocular stereo principle, is matched with OVNet to form a complete target detection and relative position estimation system for UAVs;
- The proposed pose estimation and target perception system is parallelized and accelerated using CUDA, enabling it to run in real-time on the embedded hardware platform in a real-world UAV.

## 2. Owl-Inspired Pose Estimation System

This section proposes a novel bionic simultaneous localization and mapping (SLAM) approach, inspired by the biological perception mechanisms in owls. It employs an infrared stereo camera and the IMU to simulate an owl’s eyes and vestibular system, respectively. The method integrates corner and line features from infrared images and incorporates a point–line fusion closed-loop detection module to mimic an owl’s position recognition capabilities. Initially, infrared images undergo low-light enhancement, followed by point and line feature extraction. Subsequently, frame-by-frame point–line feature matching is executed to obtain approximate camera poses and corresponding keyframes. A bag-of-words model based on point–line feature fusion is then constructed, utilizing point–line feature vocabularies for closed-loop detection of the current keyframe. When the closed-loop detection module identifies similar keyframes, the UAV is automatically relocalized. Finally, by constructing a pose graph (PG), the UAV’s pose estimation process is modeled as a nonlinear optimization problem to generate precise pose information through global optimization.

### 2.1. Visual Feature Extraction and Optimization

#### 2.1.1. Uniform ORB Feature Points

With the continuous advancement in computer vision technology, numerous image feature point detection algorithms have emerged. Renowned corner detection algorithms, such as SIFT [21] and SURF [22], have been widely applied in regard to image processing. However, due to their high computational complexity, these algorithms struggle to meet the real-time requirements of UAV pose estimation systems, even with GPU-accelerated parallelization. Conversely, algorithms with lower computational complexity, like BRIEF [23] and LDB [24], lack rotational invariance, which means that they fail to meet the basic requirements of UAV visual odometry. Therefore, this study employs the ORB [25] point feature, which is more suitable for UAV pose estimation. This method combines FAST [26] features with binary BRIEF feature descriptions, enabling ORB corners to achieve extremely rapid feature detection and matching speeds.

**Figure 3:**
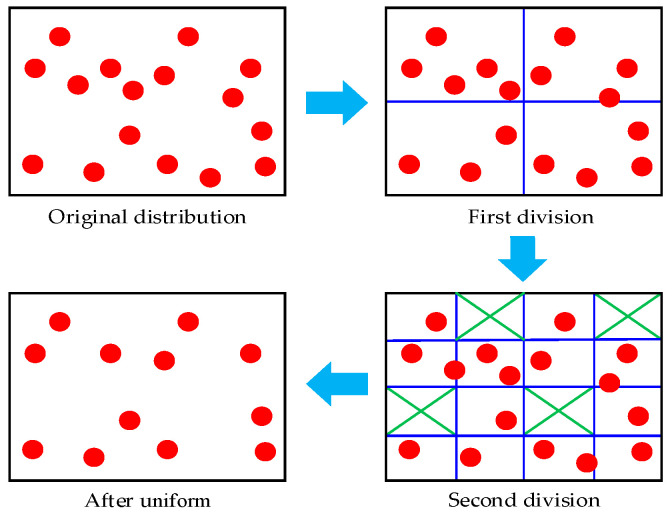
ORB feature homogenization strategy based on quadtree structure.

When visual feature points in an image are overly concentrated, the feature triangulation process becomes exceptionally challenging. Therefore, this study improves the ORB [25] corner extraction step by implementing a quadtree-based feature point uniformization algorithm. This enhancement ensures that the feature points are evenly distributed across the image, thereby improving the accuracy of stereo feature matching. Figure 3 is an illustrated flowchart of the standard quadtree algorithm, involving the specific image feature point uniformization process as follows:

1. Determine the initial number of nodes based on the aspect ratio of the input image, serving as the root node of the quadtree;
2. After the first quadrisection of the root node, it becomes four child nodes. The areas of the child nodes are divided according to the dimensions of the input image, thus determining the boundary coordinates;
3. For each node area, count the number of ORB corners. If a node area contains no ORB corners, eliminate that node. If a node contains only one ORB corner, halt its division;
4. If the total number of nodes has not reached the preset feature point threshold *n*, prioritize the division of nodes containing more feature points. If the number of nodes exceeds the threshold, select the ORB corner with the highest response value within that area as the optimal feature point for that node, while deleting other feature points in the node area.

#### 2.1.2. Line Feature Optimization

Lines in three-dimensional space have four degrees of freedom and can be parameterized with Plücker coordinates or the orthogonal representation method [27]. Let a line, ***L***, pass through point $P_{1}=\left(x_{1},y_{1},z_{1},w_{1}\right)$ and $P_{2}=\left(x_{2},y_{2},z_{2},w_{2}\right)$, so that line ***L*** can be described by a six-dimensional vector.

Although simple and intuitive in terms of projection transformation, a Plücker coordinate is excessive in terms of the parameters required and computationally expensive, with the constraint condition $n^{T}v=0$. Four-parameter orthogonal representation is more suitable for back-end optimization. The orthogonal matrix ***U***, which represents the rotation matrix that goes from the camera coordinate system to the linear coordinate system, can be decomposed by the QR decomposition:

(1) $$[n|v]=[\begin{array}{ccc} \frac{n}{\left\|n\right\|} & \;\frac{v}{\left\|v\right\|} & \;\frac{n\times v}{\left\|n\times v\right\|} \end{array}]\left[\begin{array}{cc} n & 0\\ 0 & v\\ 0 & 0 \end{array}\right]=U\left[\begin{array}{cc} n & 0\\ 0 & v\\ 0 & 0 \end{array}\right]$$

Then $\left(n,v\right)$ can be parameterized using the trigonometric function matrix, as:

(2) $$W=\left[\begin{array}{cc} w_{1} & -w_{2}\\ w_{2} & w_{1} \end{array}\right]=\left[\begin{array}{cc} \cos\left(\phi \right) & -\sin\left(\phi \right)\\ \sin\left(\phi \right) & \cos\left(\phi \right) \end{array}\right]=\frac{1}{\sqrt{\left(n^{2}+v^{2}\right)}}\left[\begin{array}{cc} n & -v\\ v & n \end{array}\right]$$

So that the orthogonal representation can be expressed as:

(3) $$\left(\begin{array}{cc} U & W \end{array}\right)\in SO\left(3\right)\times SO\left(2\right)$$

The conversion from orthogonal representation to Plücker coordinates, with $u_{i}$ denoting the *i*-th column of L′ can be expressed as:

(4) $$L\prime ={\left[w_{1}u_{1}^{T},w_{2}u_{2}^{T}\right]}^{T}=\frac{1}{\sqrt{\left(n^{2}+v^{2}\right)}}L$$

In this article, Plücker coordinates are employed during line extraction and the projection transformation process, while orthogonal representation is applied for optimization.

#### 2.1.3. Stereo Matching

Let line ***L*** pass through two points $P_{1}=(x_{1},y_{1},z_{1})$ and $P_{2}=(x_{2},y_{2},z_{2})$ in regard to spatial coordinates, then line ***L*** can be described by a six-dimensional vector:

(5) $$L=\left[\begin{array}{c} P_{1}\times P_{2}\\ P_{2}-P_{1} \end{array}\right]=\left[\begin{array}{c} n\\ d \end{array}\right]$$

where ***n*** is the normal vector of line ***L*** and ***d*** is the direction vector.

Thus, the transformation relationship between the line from the world coordinate system $L_{w}$ to the camera coordinate system $L_{c}$ can be expressed as:

(6) $$L_{c}=\left[\begin{array}{c} n_{c}\\ d_{c} \end{array}\right]=T_{cw}L_{w}=\left[\begin{array}{cc} R_{cw} & t_{cw}\\ 0^{T} & 1 \end{array}\right]L_{w}$$

where $T_{cw}$ is the transformation matrix that goes from the world coordinate system to the camera coordinate system, $R_{cw}$ is the rotation matrix that goes from the world coordinate system to the camera coordinate system, and $t_{cw}$ represents the translation vector.

The projection transformation of a line from the camera coordinate system $L_{c}$ to the pixel coordinate system ${L^{\prime }}_{uv}$ can be represented by the following equation:

(7) $${L^{\prime }}_{uv}=Kn_{c}=\left[\begin{array}{ccc} f_{v} & 0 & 0\\ 0 & f_{u} & 0\\ -f_{v}c_{u} & f_{u}c_{v} & f_{u}f_{v} \end{array}\right]n_{c}$$

where $f_{u},f_{v},c_{u},c_{v}$ are the camera’s intrinsic parameters and matrix ***K*** represents the intrinsic parameter conversion of the line from the camera coordinate system to the pixel coordinate system.

**Figure 4:**
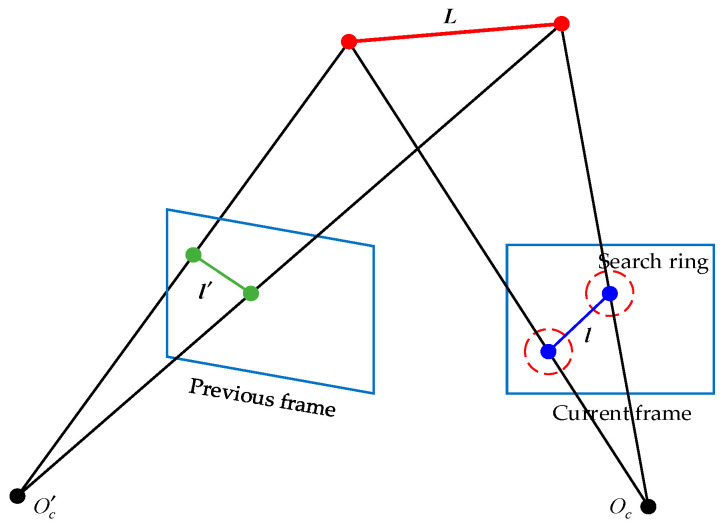
Visual feature projection matching.

After parameterizing the point and line features in three-dimensional space, the point and line features in the current power line scene before the UAV can be projected onto the image frame. First, the Liang–Barsky algorithm [28] is used to clip the line features into line segments, according to the camera’s field of view. Then, the two endpoints of the line segment are used as centers for feature searching. As shown in Figure 4, ***L*** represents a certain line segment in the current spatial environment of the UAV, $l^{\prime }$ represents the EDLines line feature of segment ***L*** in the previous image frame, and l represents the EDLines line feature to be matched in the current image frame.

### 2.2. IMU Pre-Integration

The IMU can measure the angular velocity and acceleration of three axes at a high frequency (generally not less than 200 Hz, while the camera is about 30 Hz) and the measured value contains random walk bias, which is slowly changing, as well as measurement noise, with a Gaussian character of distribution. Hence, the current position, velocity, direction, and other state values are obtained through the numerical integration of the measurements with their initial value. The state vectors of the IMU can be expressed as:

(8) $$x={\left[\begin{array}{ccccc} p & v & q & b_{g} & b_{a} \end{array}\right]}^{T}$$

where *p* denotes the position, *v* denotes the velocity, and the quaternion *q* denotes the rotation in the world coordinate system. Moreover, $b_{g}$ and $b_{a}$ represent the bias in terms of the gyroscope and the accelerometer, respectively. The observation model of the IMU can be expressed with the original IMU data, which is composed of the true value, noise, and bias, as follows:

(9) $$\begin{array}{c} {\tilde{\omega }}^{b}=\omega^{b}+b^{g}+n^{g}\\ {\tilde{a}}^{b}=q_{bw}(a^{w}+g^{w})+b^{a}+n^{a} \end{array}$$

where the superscripts *b* and *w* represent the IMU body coordinate system and world coordinate system, and *a* and *g* represent the accelerometer and gyroscope, respectively. Moreover, ${\tilde{\omega }}^{b}$ and ${\tilde{a}}^{b}$ are the measured values of the angular velocity and acceleration, and $\omega^{b}$ and $a^{w}$ are the corresponding true values. The quaternion, $q_{bw}$, represents the rotation from the world coordinate system to the IMU coordinate system. Symbol *b* and *n* represent bias and white noise. Then, the kinematic model of the IMU can be established, as follows:

(10) $$\begin{array}{c} {\dot{p}}_{b_{t}}^{w}=v_{t}^{w}\\ {\dot{v}}_{t}^{w}=a_{t}^{w}\\ {\dot{q}}_{b_{t}}^{w}=q_{b_{t}}^{w}⊗\left[\begin{array}{c} 0\\ \frac{1}{2}\omega^{b_{t}} \end{array}\right] \end{array}$$

Through adopting the observation and kinematic model of the IMU, the integration of the IMU measurements from the *i*-th time to the *j*-th time can be calculated as follows:

(11) $$\begin{array}{c} p_{b_{j}}^{w}=p_{b_{i}}^{w}+v_{t}^{w}⊗t+\iint_{t\in \left[i,j\right]}\left(q_{b_{t}}^{w}a^{b_{t}}-g^{w}\right)\delta t^{2}\\ v_{j}^{w}=v_{i}^{w}+\int_{t\in \left[i,j\right]}\left(q_{b_{t}}^{w}a^{b_{t}}-g^{w}\right)\delta t\\ q_{wb_{j}}=\int_{t\in \left[i,j\right]}q_{wb_{t}}⊗\left[\begin{array}{c} 0\\ \frac{1}{2}\omega^{b_{t}} \end{array}\right]\delta t \end{array}$$

The problem related to the asynchronous measurement frequency should be solved first, when integrating the visual and IMU data, according to which the output frequency of the IMU measurement is much higher than that of the camera and other visual sensors. IMU pre-integration refers to the integration of the IMU observation data between two moments, in advance, which was first proposed by Lupton et al. [29] to avoid calculations involving a tremendous amount of integrals caused by iterative pose optimization, depending on the initial values. Foster et al. [30] built a complete theoretical system through applying IMU pre-integration to Lie algebra. IMU pre-integration can be obtained from the IMU integration model:

(12) $$\begin{array}{c} \alpha_{b_{i}b_{j}}=\iint_{t\in \left[i,j\right]}(q_{b_{i}b_{t}}a^{b_{t}})\delta t^{2}\\ \beta_{b_{i}b_{j}}=\int_{t\in \left[i,j\right]}(q_{b_{i}b_{t}}a^{b_{t}})\delta t\\ q_{b_{i}b_{j}}=\int_{t\in \left[i,j\right]}q_{b_{i}b_{t}}⊗\left[\begin{array}{c} 0\\ \frac{1}{2}\omega^{b_{t}} \end{array}\right]\delta t \end{array}$$

With a simple formula:

(13) $$q_{wb_{t}}=q_{wb_{i}}⊗q_{b_{i}b_{t}}$$

Then, the pre-integration error can be calculated by subtracting the measured value from the estimated value over a period of time:

(14) $${\left[\begin{array}{c} r_{p}\\ r_{q}\\ r_{v}\\ r_{ba}\\ r_{bg} \end{array}\right]}_{15\times 1}=\left[\begin{array}{c} q_{b_{i}w}\left(p_{wb_{j}}-p_{wb_{i}}-v_{i}^{w}\Delta t+\frac{1}{2}g^{w}\Delta t^{2}\right)-\alpha_{b_{i}b_{j}}\\ 2{\left[q_{b_{j}b_{i}}⊗\left(q_{b_{i}w}⊗q_{wb_{j}}\right)\right]}_{xyz}\\ q_{b_{i}w}\left(v_{j}^{w}-v_{i}^{w}+g^{w}\Delta t\right)-\beta_{b_{i}b_{j}}\\ b_{j}^{a}-b_{i}^{a}\\ b_{j}^{g}-b_{i}^{g} \end{array}\right]$$

### 2.3. Backend Optimization

#### 2.3.1. Point–Line Fusion Bag-of-Words Model

**Figure 5:**
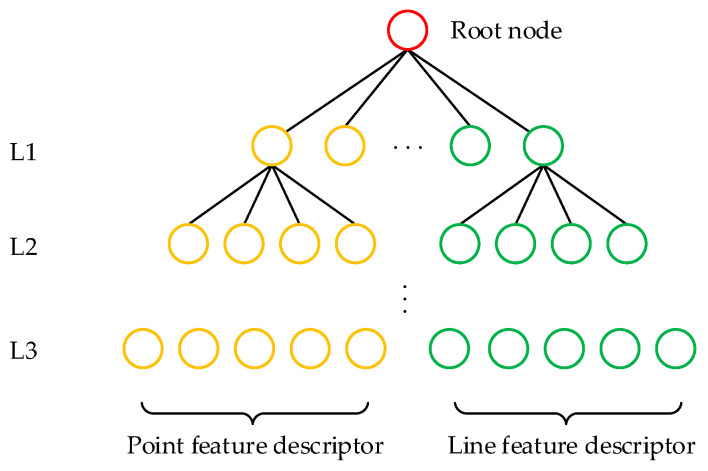
Dictionary structure diagram, based on point and line features.

To enable rapid relocalization and closed-loop detection for the operation of UAVs in power line environments, the closed-loop detection module within the proposed SLAM system utilizes the same point and line features as the system’s frontend to identify the UAV’s current pose within power line environments. To address factors that may affect closed-loop detection performance in real power line scenarios, such as lighting changes, corner feature deficiencies, and tree occlusions, this section implements an extension of the bag-of-words (BoWs) model [31] by integrating EDLines line features, effectively improving the accuracy of UAV relocalization. The dictionary tree structure based on point and line features is shown in Figure 5, where the leaf nodes in the dictionary tree are word nodes. Let the bag-of-words vectors in the two image frames be $v_{1}$ and $v_{2}$, respectively. The similarity between these two image frames can then be defined as:

(15) $$s(v_{1},v_{2})=1-\frac{1}{2}|\frac{v_{1}}{\left|v_{1}\right|}-\frac{v_{2}}{\left|v_{2}\right|}|$$

To further enhance the spatial correlation between the words in the dictionary tree, this section proposes a BoWs model based on point–line feature fusion. According to the point–line feature dictionary, we can obtain the BoWs vector $\left[v_{p_{1}},\ldots ,v_{p_{m}}\right]$ describing the point features and the BoWs vector $\left[v_{l_{1}},\ldots ,v_{l_{n}}\right]$ describing the line features for the current UAV keyframe, as well as the BoWs vector $\left[w_{p_{1}},\ldots ,w_{{p^{\prime }}_{m}}\right]$ representing the point features and the BoWs vector $\left[w_{l_{1}},\ldots ,w_{{l^{\prime }}_{n}}\right]$ representing the line features in the keyframe $K^{\prime }$ to be detected. The point and line feature similarities in the image can be expressed as:

(16) $$\left\{\begin{array}{l} s_{p}=-\frac{1}{2}\sum_{i,j}\left(\left|v_{p_{i}}-w_{p_{j}}\right|-\left|v_{p_{i}}\right|-\left|w_{p_{j}}\right|\right)\\ s_{l}=-\frac{1}{2}\sum_{i,j}\left(\left|v_{l_{i}}-w_{l_{j}}\right|-\left|v_{l_{i}}\right|-\left|w_{l_{j}}\right|\right) \end{array}\right.$$

where $s_{p}$ represents the point feature similarity score and $s_{l}$ represents the line feature similarity score. Then, based on the number of point features, $N_{p}$, and the number of line features, $N_{l}$, in the image, the image similarity score based on point–line fusion can be defined as:

(17) $$s=\frac{N_{p}s_{p}+N_{l}s_{l}}{N_{p}+N_{l}}$$

Thus, during the loop-closure detection process, by retrieving the keyframe with the highest similarity value from all the keyframes in the database, candidate loop-closure detection keyframes can be obtained.

#### 2.3.2. Loop and Map Merging

The essence of loop detection is to determine whether there is a correlation between the current frame and the earlier frame. The bag-of-words model [31], being one of the most widely used loop detection methods, is capable of judging the similarity between images by vectorizing the feature points extracted from images into visual words and establishing a vocabulary tree. A new vocabulary tree combining both point and line features is introduced to calculate the similarities between the candidate keyframes stored in the keyframe database during loop closing and relocation. The trained descriptors of the ORB point features and EDLines features are put into the same visual dictionary, then divided into the point group and line group. The new vocabulary tree structure is finally constructed through the hierarchical K-means clustering process, carried out for both the point and line feature descriptors.

**Figure 6:**
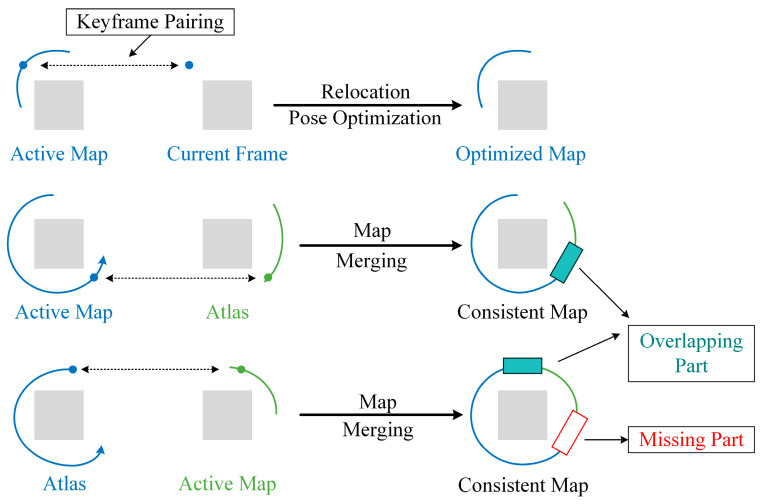
Illustration of map merging process.

A multi-map system, with map reuse and map merging, is developed to improve the integrity of the global map and the robustness of long-term tracking, as shown in Figure 6. Every inserted keyframe is trying to relocate the Atlas using multiple verifications to accurately detect whether there is a duplicated area. If the matching keyframe is in the active map, a loop is determined and a global BA is carried out to optimize the pose and map; otherwise, the data association in terms of the multi-map is implemented and the current active map is merged with the matching map. Map merging is split into several steps, so the process might take a long time. First, a welding window, defined by the matched keyframes and their covisible keyframes, is assembled. During the second step, the matched maps are fused into the new active map, with redundant points and lines being removed. Then, a local BA is performed to optimize all the keyframes in the welding window, which can be used for tracking once they have finished the optimization. Last, but not least, pose–graph optimization is implemented to propagate the corrections to the rest of the merged map.

## 3. Owl-Inspired Target Perception System

### 3.1. Designing Efficient CNNs for Real-Time Object Detection

The object detection module is a crucial component of the environmental perception system for power line inspection UAVs. It aims to search for all objects of interest within image frames, predicting their bounding boxes and categories, thereby enabling UAVs to make decisions based on the task requirements. Although numerous object detectors have been applied to specific engineering projects, such as the YOLO series [32,33,34], Sparse-RCNN [35], and TSP-FCOS [36], these detectors often involve substantial computational complexity and rely heavily on large GPUs, making it challenging to meet the real-time requirements of mobile robots and, thus, limiting their applicability.

**Figure 7:**
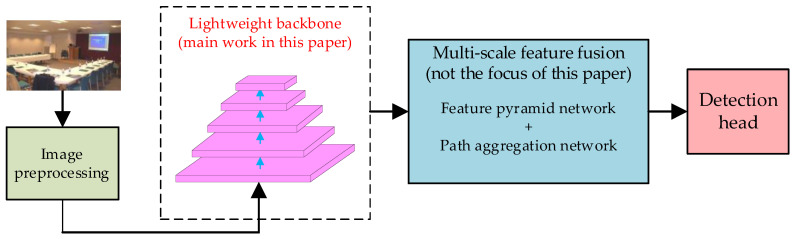
Overall architecture of the object detector.

This section mainly introduces the network architecture and explains the design of some efficient modules and tricks to make the network more suitable for deployment in embedded devices, to complete the detection of foreign objects on transmission lines. As illustrated in Figure 7, the proposed object detector framework comprises an image preprocessing module, a backbone network module, a feature fusion module, and a detection head. The image preprocessing module includes data augmentation, adaptive anchor box calculation, and image scaling operations. The backbone network is responsible for multi-scale feature extraction from the input image, after which feature maps with different dimensions are sent to the feature fusion module for multi-scale feature integration. Finally, the detection head outputs prediction boxes for the predetermined targets. To further enhance the inference speed of the object detector and enable its deployment on edge computing devices in small UAVs, this section integrates bionic mechanisms by emulating the visual neural systems of insects. We attempt to redesign a lightweight bionic neural network, OVNet, to replace the original CSPNet backbone in the TF-YOLO model, in order to enable the improved lightweight bionic object detection system to operate in real-time on small edge computing devices.

The subsequent research in this section primarily focuses on the design of the lightweight backbone network, OVNet. Each subsection proposes a new improvement measure for the original CSPNet backbone in the TF-YOLO model. Finally, adaptive neural architecture search technology is employed to construct the complete lightweight bionic neural network OVNet, thereby achieving a lightweight modification of the original TF-YOLO model.

The backbone network is the main part of feature extraction. The design of the residual structure allows us to design a very deep network to better extract the target features, but it also means that there is an increase in the number of parameters. Due to the limited memory and computing resources, we hope to keep the excellent feature extraction performance, while reducing the number of network parameters as much as possible, to complete the inference operation faster. YZNet [37] is a mainstream lightweight network, with high accuracy and that requires a small amount of model specifications. The YZNet network proposed in [37] includes a ghost module to generate more feature maps from a series of linear transformations, which is inexpensive to run. Corresponding ghost bottlenecks are designed to stack the ghost modules and there are two types, namely those with step size 1 and those with step size 2. A bottleneck with stride 1 stacks two ghost modules and a bottleneck with stride 2 inserts a depthwise convolution between two ghost modules and shortcut to downsample the feature map, which aims to finish the dimensionality reduction. The squeeze-and-excitation (SE) module, represented by the dotted box, indicates that some layers have added the channel attention mechanism module into the ghost bottleneck.

### 3.2. Binocular Ranging Platform

When UAVs perform contact operations involving power transmission lines, in addition to acquiring semantic information on the targets, they also need to obtain the relative positional relationship between the targets and the UAV. Therefore, based on previous team research [38,39,40,41], this article extends the target relative position estimation capability and incorporates foreign object detection and path planning algorithms. This enables UAVs involved in power operations to acquire distance information on the targets, while capturing semantic information on the targets, facilitating rapid and effective tracking.

**Figure 8:**
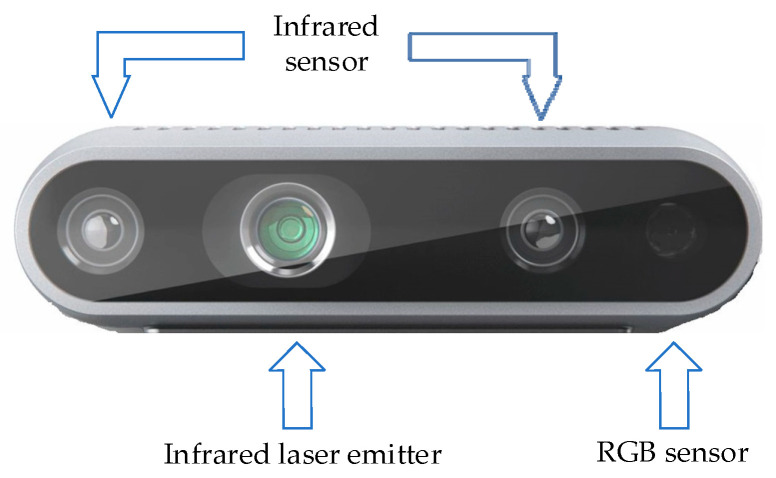
The structure of a D435i camera.

Figure 8 illustrates the structure of a D435i binocular camera, consisting of a pair of stereo infrared sensors, an infrared laser emitter, and a color camera. The left and right infrared cameras produce two grayscale images, while the central infrared dot matrix emitter serves as an infrared fill light. The rightmost RGB camera is used to capture color images, ultimately enabling the alignment of color video streams with depth streams.

**Figure 9:**
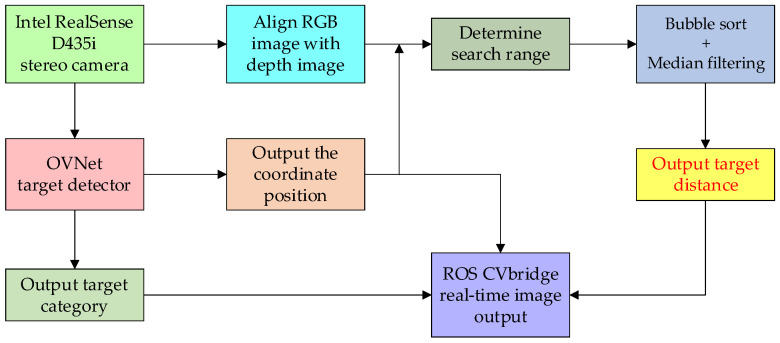
The flowchart of target measurement system.

After the binocular camera is activated for original image acquisition, the ranging algorithm process occurs, as shown in Figure 9. The RGB image is processed and transmitted to the pre-trained OVNet power line foreign object detection model. By forwarding the input image through the network, the output includes target categories and foreign object coordinate positions in the image. The foreign object coordinate position information, in addition to being used for the final output of the rectangular box construction, is also transmitted to the ranging section.

Another operational pathway in terms of the binocular camera involves aligning the corresponding RGB image and the depth map of the original image. Subsequently, combining the foreign object position coordinate information obtained from the foreign object detection network, the central pixel position for depth indexing is determined, establishing the deviation range for the depth search. At this point, a random sequence of length 40 is declared, with each sequence number for the same target box generating a coordinate corresponding to a depth value. The 40 depth values are then placed in a list for bubble sorting, followed by median filtering, ensuring that the remaining depth values are essentially reliable and truly reflect the depth between the foreign object and the camera. Finally, the average of the remaining depth values is calculated to obtain the actual distance between the detected foreign object within the target box and the camera.

### 3.3. Owl-Inspired Target Tracking System

The bionic target tracking system designed in this article primarily consists of an object detection module, a target ranging module, an obstacle recognition module, and a path planner. These components simulate an owl’s visual recognition and ranging system, as well as its optical flow tracking and obstacle avoidance system. All the functional modules collaborate to guide the UAV through the target tracking process. The object detection module can directly utilize the OVNet–YOLO model proposed in Section 3.1 of this article. Given that existing obstacle recognition modules and UAV path planners are already highly mature, this section directly employs the original algorithms embedded in the ROS to implement the UAV’s autonomous obstacle avoidance functionality. Although numerous algorithms for target ranging currently exist, these ranging algorithms often lack pattern recognition capabilities. Algorithms that simultaneously address UAV target detection and relative position estimation are still at the theoretical stage. Therefore, this section focuses on the need to redesign a complete target tracking system specifically for small mobile UAVs.

**Figure 10:**
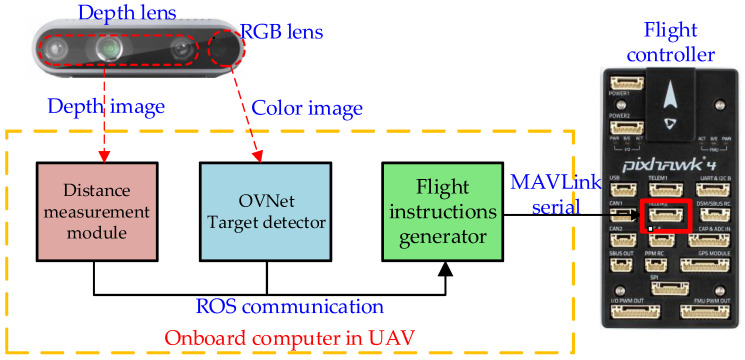
Diagram of the owl-inspired target tracking system.

The input into the OVNet object detection module is a two-dimensional image, which lacks the ability to perceive the relative distance between the UAV and the target. When UAVs perform target tracking tasks, in addition to acquiring semantic information on the target to be tracked, they also need to obtain information on the relative position between the target and the UAV. Therefore, this section extends the target relative position estimation functionality based on the OVNet lightweight object detection system and incorporates obstacle recognition and path planning algorithms. This enables small UAVs to acquire distance information on the targets, while capturing semantic information on the targets, facilitating rapid and effective tracking. We employ a mounted Intel RealSense D435i stereo camera as the visual perception source. The color image from the D435i camera is fed into OVNet for pattern recognition, while the depth image from the D435i camera is fed into the ranging module to estimate the spatial position information on the obstacles and the target to be tracked. Finally, the relative distance information on the target to be tracked and the surrounding obstacles is sent to the UAV path planner, which guides the UAV to complete target tracking. Figure 10 presents the overall design framework of the owl-inspired target tracking system.

## 4. Experiments

### 4.1. Performance Evaluation of Pose Estimation Algorithm

**Figure 11:**
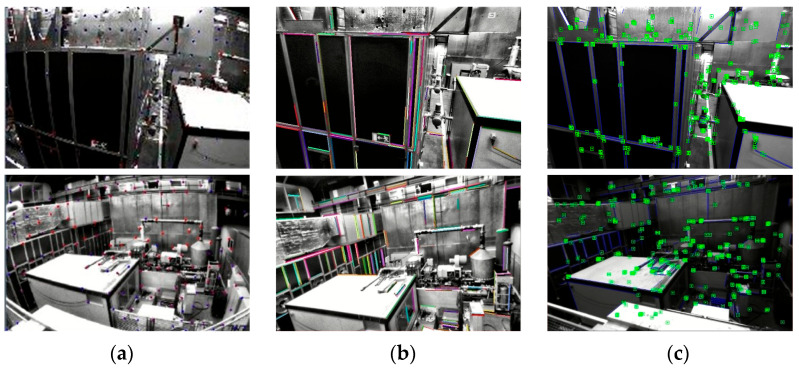
A comparison between the feature extraction performance of the VINS-Fusion, PL-VIO, and our proposed algorithm: (**a**) VINS-Fusion; (**b**) PL-VIO; (**c**) proposed algorithm. The dots and lines in the figure respectively represent the extracted point features and line features.

Given that the proposed pose estimation algorithm is designed to meet the autonomous navigation requirements of power line inspection UAVs, the EUROC dataset [42] was selected for the evaluation of the UAV pose estimation algorithm. The EUROC dataset was collected by a small multi-rotor UAV equipped with a stereo camera, with data acquisition conducted in an indoor factory and two laboratories. Figure 11 illustrates the visual feature extraction performance comparison between the proposed algorithm and the VINS-Fusion [43] and PL-VIO [44] algorithms using the indoor factory dataset.

Table 2 presents a comparison between the performance of the proposed algorithm with other similar algorithms using the EUROC dataset, with the system’s performance evaluated using the root mean square error of the absolute trajectory error (ATE). In regard to the V1_02 sequence, the PL-VIO algorithm was unable to continue visual feature tracking due to the UAV executing a large-angle yaw maneuver, while facing a pure white wall. By integrating EDLines line features with ORB point features, the proposed algorithm maintains smooth visual feature tracking even during intense UAV motion or large-scale yaw maneuvers.

**Figure 12:**
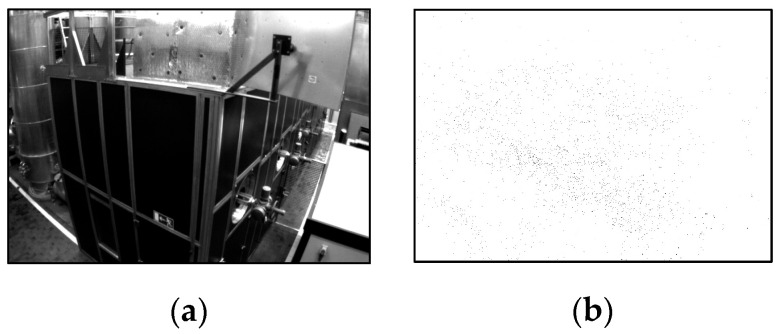

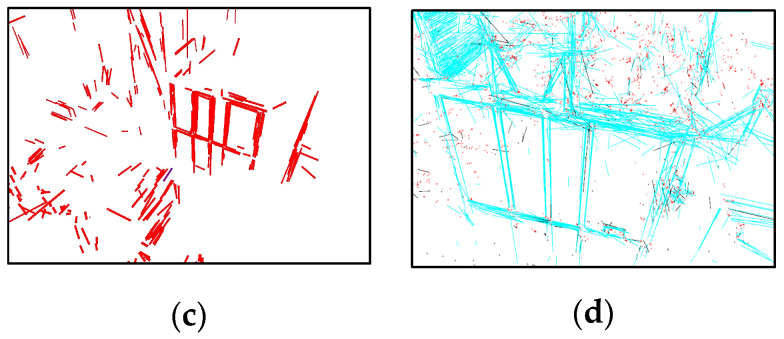
A comparison between the mapping effects of different mapping methods: (**a**) raw image; (**b**) VINS-Fusion; (**c**) PL-VIO; (**d**) proposed method.

The proposed biomimetic SLAM system also demonstrates more intuitive mapping effects compared to other similar algorithms, as shown in Figure 12. The proposed three-dimensional mapping module simultaneously constructs point and line features in the UAV environment, resulting in a map with richer environmental textures that can provide robust support for subsequent autonomous navigation and obstacle avoidance by power line inspection UAVs.

### 4.2. Evaluation of Target Perception Algorithm

This section compares the proposed target perception algorithm with widely applied object detectors, such as SSD, Faster R-CNN, YOLOv4, YOLOv5, YOLOX, and PP-YOLOE, using the LFM2021 power line foreign object dataset. The experimental results are shown in Table 3. The evaluation criteria for target perception algorithms include the mean average precision (mAP50) for each category, the frames per second (FPS), and the number of model parameters. The mAP50 represents the model’s average detection precision at an IOU threshold of 0.5, the FPS directly indicates the model’s inference speed, and the parameter count represents the number of trainable parameters in the module.

Table 3 clearly shows that the proposed algorithm’s model inference speed is nearly 200% faster than the YOLOv5 model, demonstrating unparalleled real-time performance compared to other mainstream algorithms. Moreover, with only 1.69 M parameters, it is less than one-twelfth the size of the lightweight neural network YOLOv5, offering significant energy efficiency advantages in terms of small UAV onboard computers.

To test real-world power line scenarios, in addition to performance testing using the dataset, a 10-m long suspended aluminum–iron wire was constructed for this purpose. Foreign objects, such as kites and balloons, were attached to this wire to simulate scenarios involving objects adhered to power transmission lines. The aluminum–iron wire, weighing thirty kilograms, closely approximates the specifications of high-voltage transmission lines.

**Figure 13:**
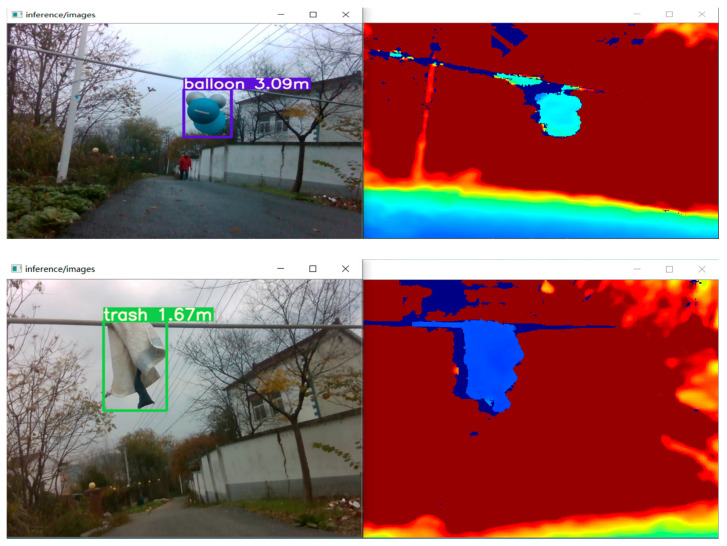
Performance-related demonstration of the target ranging system for an outdoor on-site operation.

Figure 13 demonstrates the visualization of power line target distance measurement. The left image shows the original RGB camera image after detection by the OVNet network, which identified the foreign object type and generated corresponding bounding boxes for localization. Subsequently, the stereo ranging algorithm determined the distance of the foreign object from the camera, with the detected distance information labeled after the category, which is accurate to the centimeter level. The right image displays the depth map captured by the infrared sensor of the stereo camera, revealing the depth information in the image. Objects at the same depth have similar colors, effectively highlighting the contours of the foreign objects in the image. During the target distance measurement process, OVNet’s output is smooth, meeting the basic real-time requirements for environmental perception of power line inspection UAVs.

### 4.3. UAV Autonomous Attachment Test

**Figure 14:**
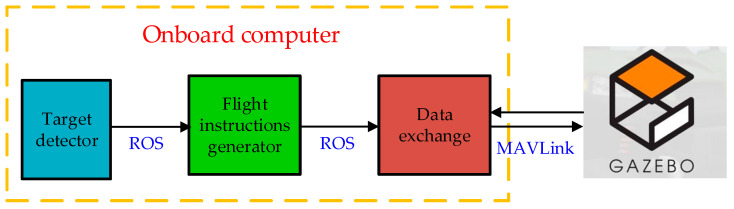
Logic diagram for the UAV autonomous attachment experiment.

To further validate the practical application value of the proposed algorithm, this section utilizes the Gazebo simulator to construct an experimental scenario for autonomous power line attachment by a small quadcopter UAV. After completing the attachment to the power transmission line, the UAV can shut down its rotors and use the line itself as a point of contact to perform power maintenance tasks. Figure 14 illustrates the code logic relationship for the UAV autonomous power line attachment experiment.

**Figure 15:**
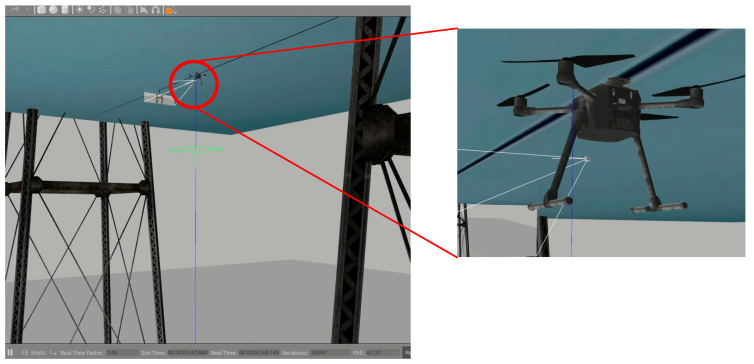
Autonomous attachment in Gazebo simulation environment.

First, after takeoff, the UAV sends RGB images from the upward-facing camera to the environmental perception module for power line detection. When power lines appear in the upward-facing camera’s field of view, the object detector calculates the relative yaw angle between the UAV and the power line. The flight command generator then issues yaw commands to the UAV based on the current relative yaw angle, aiming to reduce the yaw angle between the UAV and the power line to zero. Subsequently, the power line inspection UAV ascends at a preset constant speed. When the locking mechanism located above the UAV body contacts the power line, the UAV shuts down its rotor motors, completing its attachment to the power line. Figure 15 shows the simulated scenario involving the power line inspection UAV completing automatic attachment to the power line.

### 4.4. Power Line Foreign Object Detection in the Real World

To further validate the practical application value of the proposed algorithm, this section employs a small multi-rotor UAV to conduct the on-site inspection of actual power transmission lines. The experimental aircraft chosen is a quadrotor UAV with a 410 mm wheelbase, using Pixhawk4 autopilot as the flight control platform and an NVIDIA Jetson NX embedded computer as the onboard computer. The assembled prototype UAV used for the physical experiment is shown in Figure 2b. The UAV inspection process was completed under the on-site guidance of professional personnel from the power supply bureau.

**Figure 16:**
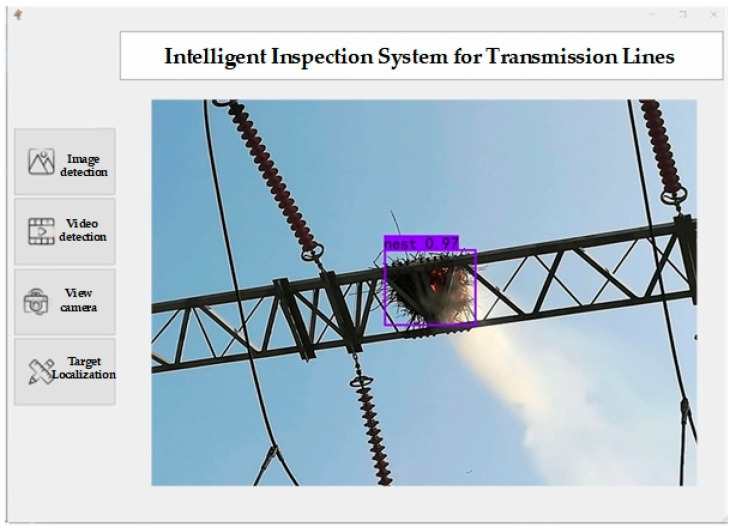
The GUI of power line foreign object detection system.

After the UAV flies above the high-voltage transmission lines, clicking the “View camera” button on the ground control terminal activates the UAV camera. Subsequently, the environmental perception module is activated for foreign object detection, as shown in Figure 16. If there is only one camera, the default number is “0”; if multiple cameras are connected externally, the number needs to be adjusted to correspond to the specified camera number, otherwise an error window will pop up displaying “Failed to open camera”. When the camera is closed, the display interface reverts to the background image. After detection is complete, the entire detection process is saved as a video file named “trans.avi” in the corresponding folder; information is recorded from the camera’s activation to its termination. This facilitates the recording of the entire UAV inspection process, for subsequent review by power maintenance personnel.

## 5. Conclusions and Future Work

We utilized infrared stereo cameras and the IMU to simulate the eyes and vestibular organ of nocturnal owls, achieving self-pose estimation of transmission line maintenance UAVs. By integrating corner features and line features in power line scenarios and employing a loop-closure detection module, based on point–line fusion, for power line inspection UAV relocalization, a novel biomimetic SLAM method is proposed. Concurrently, a compatible target perception system is designed. This perception system employs a lightweight biomimetic neural network to mimic an owl’s visual nervous system, performing object detection involving the onboard camera’s output, and uses a binocular ranging algorithm to simulate the binocular spatial perception mechanism of animals, obtaining the relative position relationship between the targets and the UAV. Compared to traditional robot SLAM methods, the proposed algorithm efficiently extracts point and line features from infrared images, demonstrating superior robustness in power line environments.

The experimental results indicate that, in terms of the pose estimation performance of unmanned aerial vehicles, the proposed algorithm improves the positioning accuracy by 53.3% compared to traditional methods. For object detection tasks, the proposed OVNet model improves the inference speed by 110.6% compared to the original model. The proposed biomimetic pose estimation and target perception method achieves desirable outcomes in terms of both pose estimation accuracy and the system’s operational robustness in real-world scenarios.

Although the algorithm proposed in this article was verified to a certain extent using physical experiments, we still see multiple potential areas for improvement for future work. The environmental perception of UAVs is a very complex task that involves cross-disciplinary knowledge from various disciplines. Simple object detection and relative position estimation cannot meet all the environmental perception needs in relation to transmission line operation tasks. We plan to design a new fusion strategy for magnetometer and navigation satellite sensors in future work to further improve the performance of the UAV navigation system. Furthermore, how to accurately generate a semantic map around a UAV is additional research we will carry out in the future.

## Figures and Tables

**Table 1:** Comparison of the advantages and disadvantages of foreign object removal strategies.

Strategies	Advantages	Limitations
Performed by humans	High reliability	Inefficiency and high cost
Performed by robots	High efficiency	Reliant on skilled operators
Proposed	Autonomous and unmanned	Low reliability

**Table 2:** A comparison between the performance of the proposed algorithm and other similar algorithms (in meters).

Sequence	PL-SLAM	VINS-Fusion	PL-VIO	Proposed
MH_01	0.168	0.163	0.158	0.058
MH_02	0.088	0.115	0.140	0.034
MH_03	0.100	0.193	0.269	0.138
MH_04	0.222	0.225	0.362	0.064
MH_05	0.245	0.204	0.271	0.084
V1_01	0.055	0.115	0.075	0.029
V1_02	0.109	0.087	-	0.060
V1_03	0.169	0.097	0.195	0.048
V2_01	0.073	0.086	0.093	0.073
V2_02	0.100	0.138	0.155	0.057
V2_03	0.198	0.216	0.312	0.069

**Table 3:** A comparison of the performance of different object detectors.

Target Detector	mAP50/(%)	FPS (Titan)	Parameters
SSD	89.54	87.2	24.15 M
Faster R-CNN	90.25	13.9	136.77 M
YOLOv4	95.70	46.7	63.95 M
YOLOv5	95.30	58.8	21.05 M
YOLOX	93.70	77.1	8.94 M
PP-YOLOEs	96.1	124.7	7.93 M
OVNet	95.50	162.4	1.69 M

## Data Availability

The EUROC dataset: https://projects.asl.ethz.ch/datasets/doku.php?id=kmavvisualinertialdatasets (accessed on 28 September 2024).
